# Olanzapine‐induced cardiomyopathy: A mimicker of obesity cardiomyopathy?

**DOI:** 10.1002/clc.24278

**Published:** 2024-05-20

**Authors:** Oluwaremilekun Zeth Tolu‐Akinnawo, Toluwalase Awoyemi, Rocio Barriga Guzman, Akhtar Naveed

**Affiliations:** ^1^ Piedmont Augusta Augusta USA; ^2^ Northwestern University Feinberg School of Medicine Chicago USA; ^3^ Advocate Illinois Masonic Medical Center Chicago USA

**Keywords:** cardiomyopathy, olanzapine

## Abstract

Olanzapine, an atypical antipsychotic medication, has gained prominence in the treatment of schizophrenia and related psychotic disorders due to its effectiveness and perceived safety profile. However, emerging evidence suggests a potential link between olanzapine use and adverse cardiovascular effects, including cardiomyopathy. This narrative review explores the mechanisms, clinical implications, and management strategies associated with olanzapine‐induced cardiomyopathy. A comprehensive review of the literature was conducted to investigate the relationship between olanzapine and cardiomyopathy. The search included epidemiological studies, clinical case reports, and mechanistic research focusing on the pathophysiology of olanzapine‐induced cardiomyopathy. The review also examined treatment strategies for managing this potential complication. Olanzapine‐induced cardiomyopathy is hypothesized to be associated with metabolic disturbances and receptor antagonism. The metabolic effects of olanzapine, such as weight gain, insulin resistance, and dyslipidemia, share similarities with obesity‐related cardiomyopathy. Additionally, olanzapine's antagonism of certain receptors may contribute to cardiovascular stress. The review highlighted that patients with new‐onset heart failure and significant weight gain while on olanzapine should be closely monitored for signs of cardiomyopathy. Early detection and prompt withdrawal of olanzapine, along with initiation of goal‐directed medical therapy, are crucial for mitigating this potentially life‐threatening condition. The relationship between olanzapine and cardiomyopathy is complex and not yet fully understood. However, the potential for significant cardiovascular risk necessitates vigilance among healthcare providers. Early identification and management of olanzapine‐induced cardiomyopathy can improve patient outcomes. Further research is needed to elucidate the precise mechanisms behind this adverse effect and to develop optimized treatment strategies for patients requiring antipsychotic therapy.

## 1 INTRODUCTION

The advent of antipsychotic medications in the 1950s represented a significant turning point in the treatment of schizophrenia and other psychotic disorders, thereby introducing a new era of therapeutic possibilities.^1^ Despite notable advancements in managing these conditions, the efficacy of antipsychotics is often limited by their adverse effects.^1^ Cardiomyopathy is a rare but reported complication of antipsychotic medications, with a few cases documented in the literature.^2^, ^3^ Although clozapine‐induced cardiomyopathy has been more frequently reported, there is a risk of myocarditis associated with first‐generation antipsychotics such as haloperidol and fluphenazine.^2^, ^4^ Olanzapine, an atypical antipsychotic belonging to the thienobenzodiazepine class, has gained widespread use owing to its perceived safety compared to its predecessor, clozapine.^1^, ^2^ In recent years, olanzapine has seen an increase in its use for treating negative psychiatric symptoms due to its more favorable side effects profile compared to first‐generation antipsychotics, with a lower risk of extrapyramidal side effects. However, olanzapine has the potential to cause life‐threatening hematological side effects such as agranulocytosis.^2^, ^5^ It is commonly prescribed for the treatment of multidrug‐resistant schizophrenia, acute severe agitation, and behavioral bipolar disorder.

There is growing concern regarding the potential association between olanzapine use and adverse cardiovascular outcomes, particularly cardiomyopathy and conduction abnormalities.^2^, ^4^, ^6^ Although the precise mechanisms underlying olanzapine‐induced cardiomyopathy remain undetermined, hypotheses implicate metabolic disturbances such as dyslipidemia, insulin resistance, and antagonist effects on various receptors, including histamine and adrenergic receptors.^5^, ^7^, ^8^, ^9^ Furthermore, olanzapine has been associated with severe heart and muscle conditions, including myocarditis and rhabdomyolysis.^3^ Although olanzapine is commonly considered safe about corrected QT interval, it still poses risks related to weight gain and cardiovascular health.^10^ There have been reported cases of olanzapine‐inducing myocarditis and rhabdomyolysis, even after a considerable time following the initiation of treatment.^3^

Available evidence suggests that the incidence of olanzapine‐induced cardiomyopathy is relatively low. However, individual patient factors, such as pre‐existing cardiovascular conditions, may contribute to its development.^1^, ^11^ Despite the unclear mechanism, metabolic disturbances resulting from weight gain have been proposed as a potential pathway for olanzapine‐induced cardiomyopathy, as weight gain is an independent risk factor for cardiovascular complications.^12^ In a study of 80 patients taking olanzapine, 60 completed the study, and 66.6% experienced a weight gain of 1−5 kg over 4 weeks.^1^ Notably, the incidence of olanzapine‐induced weight gain was higher in females (60%) than males (40%).

Interestingly, the dose of the drug was not correlated with the incidence of weight gain. These findings are consistent with those of previous studies.^8^, ^13^, ^14^, ^15^ The prevalence of olanzapine‐induced cardiomyopathy is challenging to determine due to the scarcity of large‐scale, prospective studies.^10^, ^11^ This article provides a comprehensive review of olanzapine‐induced cardiomyopathy, highlighting its potential resemblance to obesity‐related cardiomyopathy.

## 2 REVIEW AND DISCUSSION

### 2.1 Symptoms of olanzapine‐induced cardiomyopathy

The administration of olanzapine may result in the emergence of cardiomyopathy, a condition that can present with several symptoms, including chest discomfort, shortness of breath, generalized edema, and signs of heart failure (HF), such as reduced ejection fraction and enlarged heart chambers.^16^ Patients who are prescribed olanzapine may commonly experience adverse effects such as weight gain, diabetes, elevated lipid levels, and a lengthened QT interval.^16^ Although the development of cardiomyopathy due to olanzapine is rare, it is crucial to consider this possibility in patients experiencing cardiac symptoms while on medication. Prompt recognition, cessation of the drug, and effective management of HF are critical for preventing long‐term damage to the heart muscle in such cases.^16^ Continuous monitoring of patients taking olanzapine for potential cardiovascular complications is paramount to ensuring prompt intervention and care.

### 2.2 Olanzapine use, weight gain, and cardiovascular hemodynamics

Research has established a dose‐dependent relationship between HF and body mass index (BMI), with a 2016 meta‐analysis revealing a 41% increase in HF risk for every 5 kg/m^2^ increase in BMI.^17^ This association involves several factors, including heightened cardiac stress, altered hemodynamics, sympathetic drive, and elevated risks of concurrent cardiovascular conditions such as diabetes, hypertension, hyperlipidemia, and sleep apnea.

The pathophysiology of cardiomyopathy in patients receiving olanzapine therapy is rooted in the heart's challenge to meet metabolic demands despite a higher‐than‐normal cardiac output (CO), termed “metabolic cardiomyopathy.”^18^ In obese individuals, CO can reach levels as high as 10 L/min, compared to 5−6 L/min in those with average weight.^19^ Long‐term implications of metabolic cardiomyopathy include cardiac remodeling marked by left ventricular (LV) hypertrophy, cardiac fibrosis, and diastolic dysfunction, ultimately leading to HF. The abnormal accumulation of fat, as observed in olanzapine use, drives increased CO and workload, resulting in disproportionate LV hypertrophy to meet energy and metabolic demands, particularly evident in patients with chronic olanzapine use.^20^ This aberrant fat deposit also impacts the heart's contractility, relaxation, and survival, potentially leading to HF.^21^, ^22^

Similar to obesity‐induced cardiomyopathy, olanzapine‐induced cardiomyopathy can manifest as systolic, diastolic, or combined HF.^23^, ^24^ Additionally, individuals using olanzapine are more likely to be obese, predisposing them to comorbidities such as hypertension, sleep apnea, or diabetes mellitus. These conditions, in turn, increase the risk of anatomic and morphological myocardial pathologies, including myocardial infarction, arrhythmias, and HF.^25^

**Figure 1:**
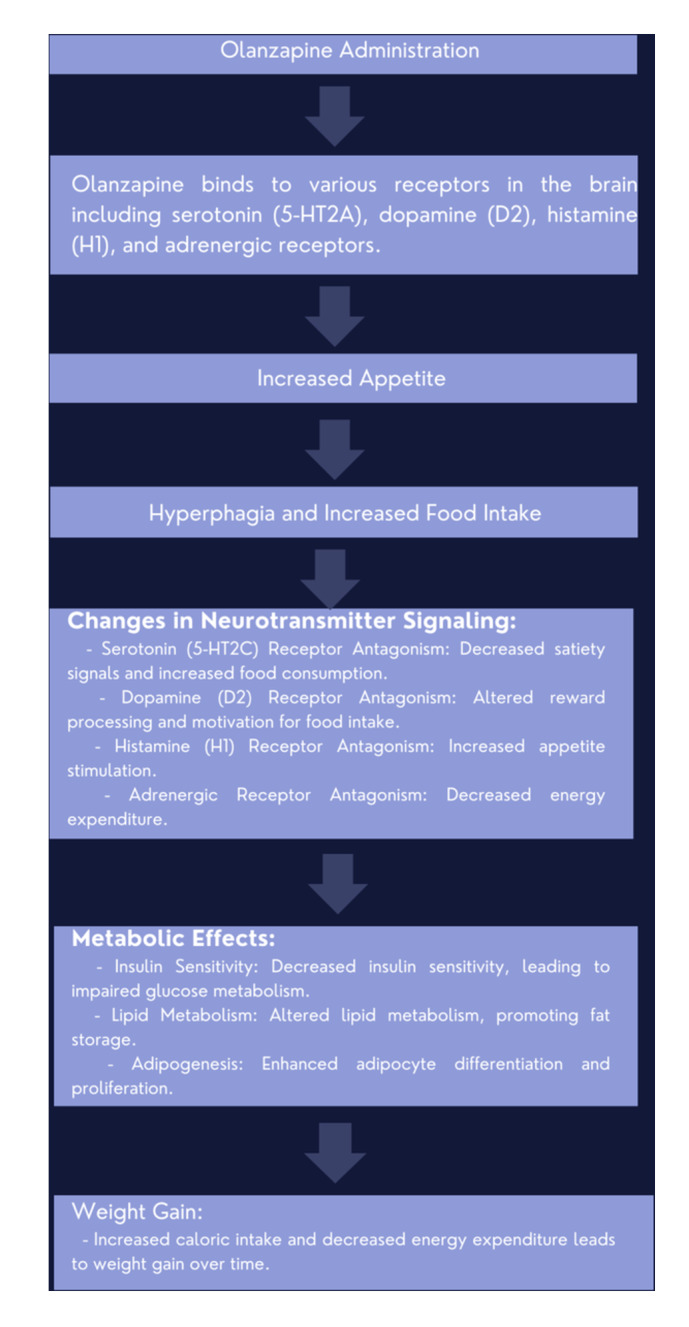
Mechanisms involved in olanzapine‐induced weight gain.

Figure 1 outlines the proposed steps in the mechanism of weight gain associated with olanzapine use, including its effect on appetite, neurotransmitter signaling, and metabolic processes, ultimately resulting in increased calorie intake and decreased energy expenditure, leading to weight gain.

### 2.3 Hemodynamic alterations driving HF in chronic olanzapine use include

#### 2.3.1 Elevated blood pressure

Olanzapine has been linked to obesity, a primary risk factor for hypertension,^26^, ^27^ although the underlying mechanism remains incompletely understood. Possible mechanisms include sodium retention, increased sympathetic stimulation, and activation of the RAAS pathway.^28^, ^29^ Metabolic syndrome, comprising hypertension, dyslipidemia, and hyperglycemia, is implicated in 45−50% of cardiovascular diseases (CVDs), including HF and myocardial infarction.^30^

#### 2.3.2 Arterial stiffness

Chronic olanzapine use results in excessive fat deposition in blood vessels, leading to decreased distensibility and increased vascular stiffness. This contributes to endothelial dysfunction, extracellular matrix remodeling, calcification, and inflammation, culminating in elevated blood pressure and increased myocardial wall stress. These changes prompt LV wall remodeling and subsequent cardiac dysfunction.^31^, ^32^, ^33^, ^34^, ^35^, ^36^

#### 2.3.3 Peripheral vascular resistance (PVR), increased CO, and blood volume

Hypertension's association with elevated peripheral vascular resistance inversely correlates with BMI, leading to increased CO.^37^ Additionally, increased blood volume exacerbates sodium retention and metabolic demands, triggering the sympathetic nervous system and RAAS activation while downregulating natriuretic peptides. These alterations impair sodium excretion and contribute to hypertension, predisposing to volume overload and high‐output HF.^17^, ^38^, ^39^, ^40^, ^41^, ^42^, ^43^

#### 2.3.4 Increased LV wall stress

Elevated blood volume, stroke volume, and CO contribute to LV wall stress, resulting in LV dilatation and eccentric hypertrophy, which leads to diastolic dysfunction. Systolic dysfunction may ensue when LV dilatation fails to compensate for excessive wall stress and thickening.^44^

#### 2.3.5 Pulmonary hypertension

The precise mechanism underlying pulmonary hypertension in chronic olanzapine use remains unclear. It may involve an interplay between increased LV filling pressure and pulmonary capillary wedge pressure typically associated with LV failure.^45^ Furthermore, the increased risk of sleep apnea and obesity hypoventilation syndrome in patients on chronic olanzapine therapy may contribute to hypoxic vasoconstriction. This process leads to right ventricular remodeling, a hallmark in developing pulmonary hypertension.^46^, ^47^, ^48^

### 2.4 Proposed mechanism of olanzapine‐induced cardiomyopathy

As previously discussed, olanzapine‐induced myopathy arises from obesity/metabolic cardiomyopathy, attributed to olanzapine's potential to induce obesity and metabolic dysregulation. This process involves a complex interplay among adipose tissue dysfunction and inflammation, metabolic disturbances, calcium handling, and autophagy.

Olanzapine initiates obesity, triggering a progressive pathological cascade characterized by activating adipocytes and resident immune cells, leading to the release of proinflammatory cytokines.^49^ In obesity, elevated levels of proinflammatory cytokines such as adiponectin, leptin, resistin, nitric oxide, interleukins, tumor necrosis factor (TNF), and other inflammatory mediators interact with systemic changes, including increased insulin resistance, RAAS activation, lipotoxicity, and interstitial fibrosis, contributing to CVD development in olanzapine‐treated patients. The primary trigger is the excess adiposity resulting from olanzapine‐induced metabolic dysregulation. Adipose tissue dysregulation and inflammation directly and indirectly exacerbate cardiomyopathy. Moreover, excessive adipose tissue deposition promotes insulin resistance, impaired glucose and lipid metabolism, and systemic inflammatory reactions, including the release of interleukins (IL‐6, 10, 13, 14), TNF, and diffuse macrophage activation. These processes may culminate in myocarditis, a common pathway observed in clozapine‐induced cardiomyopathy.^50^, ^51^, ^52^

**Figure 2:**
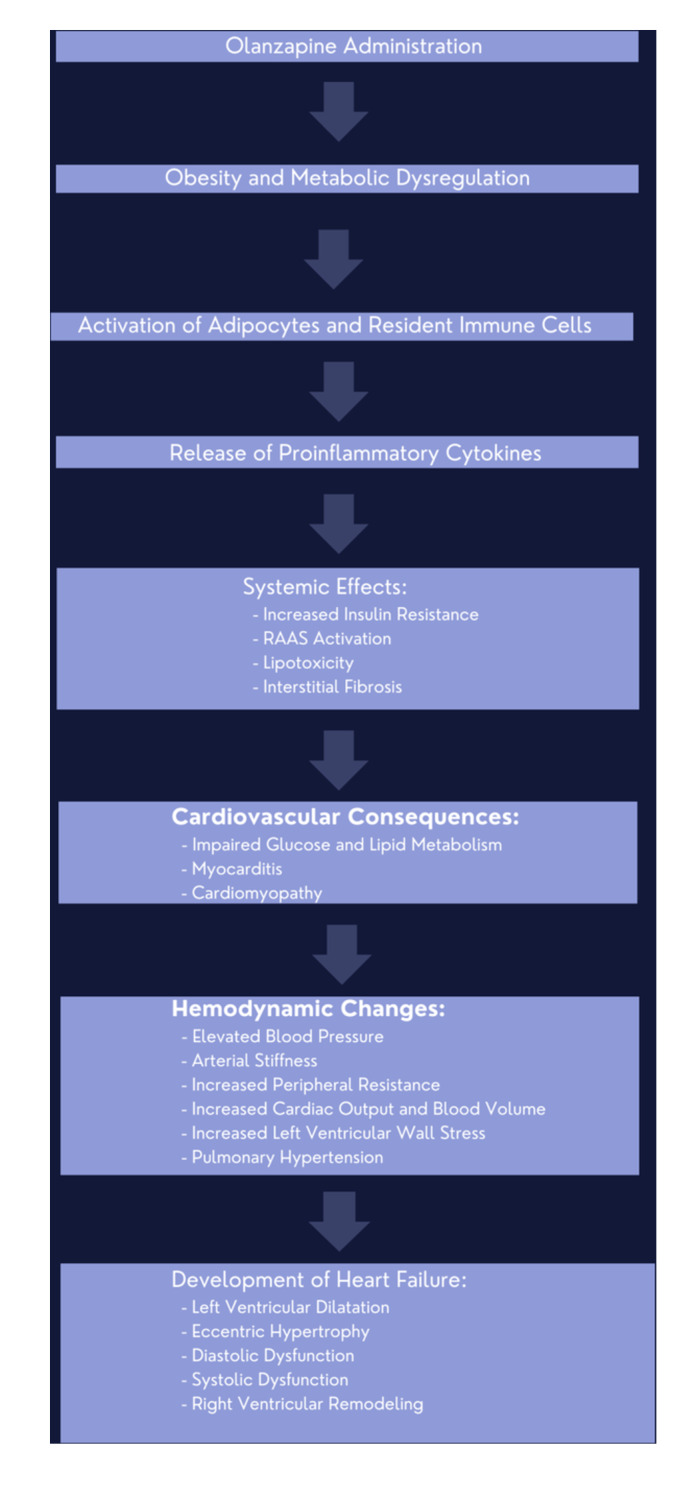
Mechanisms involved in olanzapine‐induced cardiomyopathy.

Animal studies have corroborated these findings, further supporting the role of olanzapine exposure in cardiomyopathy development.^53^ Figure 2 below provides a visual summary of the proposed mechanism of olanzapine‐induced cardiomyopathy.

### 2.5 Proposed mechanisms of olanzapine‐induced cardiomyopathy in Figure 2 below

#### 2.5.1 Timeline of olanzapine‐induced cardiomyopathy

The development of cardiomyopathy due to olanzapine can vary among individuals, as reported in the literature. For instance, a 28‐year‐old patient developed dilated cardiomyopathy after nearly a decade of olanzapine treatment,^16^ and another study found a connection between clozapine and HF within 3 years but did not establish an association between olanzapine, quetiapine, and the cardiac adverse events under investigation.^54^ Although the timeline for the onset of olanzapine‐induced cardiomyopathy has not yet been standardized, it is essential to monitor patients regularly for signs of cardiac dysfunction, particularly those receiving long‐term olanzapine therapy. Prompt identification and discontinuation of the medication are critical to prevent irreversible myocardial damage in such cases.^16^, ^55^

#### 2.5.2 Treatment of olanzapine‐induced cardiomyopathy

Managing olanzapine‐induced cardiomyopathy requires prompt recognition of the condition, cessation of medication, and effective management of symptoms of HF. Discontinuing the drug is crucial to alleviate further harm to the myocardium.^16^, ^55^ Patients diagnosed with this condition may necessitate standard HF treatment strategies, such as fluid restriction, digoxin, ACE inhibitors, β‐blockers, and diuretics.^16^ These therapeutic agents aim to improve cardiac function and alleviate symptoms associated with HF. Regular monitoring and subsequent evaluations are vital for assessing the patient's response to treatment and adapting the management regimen as needed.^16^

Furthermore, studies suggest that consistent physical activity can help counteract the adverse effects caused by olanzapine, including metabolic issues like weight gain and insulin resistance, which elevate the risk of cardiovascular complications.^56^ In addition to exercise, dietary adjustments to maintain a healthy weight and regulate metabolic indicators such as blood sugar levels and lipid profiles can prove advantageous in managing olanzapine‐induced cardiomyopathy.^56^ Embracing lifestyle modifications centered on a well‐rounded diet, weight control, and regular physical activity can complement medical interventions and enhance the prognosis for individuals grappling with this condition.^16^

## 3 CONCLUSION

In summary, olanzapine is a significant advancement in the treatment of schizophrenia and related psychiatric disorders. However, it is essential to closely monitor patients taking medication because of its potential association with cardiomyopathy, particularly in obese patients. The detection of olanzapine‐induced cardiomyopathy requires a high level of suspicion, as new‐onset HF in patients receiving olanzapine without a prior cardiac history, especially when accompanied by weight gain, should raise concerns. Recent case reports have illustrated this point.^54^ The proposed mechanisms for olanzapine‐induced cardiomyopathy include metabolic disturbances and adipose tissue dysfunction, highlighting the complexities involved. Early discontinuation of olanzapine upon suspicion has resulted in symptom improvement, and the management of HF with goal‐directed medical therapy (GDMT) is crucial for the prognosis of olanzapine‐induced cardiomyopathy. Further research is necessary to determine the optimal exposure duration, treatment strategies, and prognosis. In addition, it is essential to elucidate the precise pathophysiology and develop optimal management strategies for olanzapine‐induced cardiomyopathy.

## CONFLICT OF INTEREST STATEMENT

The authors declare no conflict of interest.

## Data Availability

The data that support the findings of this study are available from the corresponding author upon reasonable request.

## References

[1] Jain S , Bhargava M , Gautam S . Weight gain with olanzapine: drug, gender or age. Indian J Psychiatry. 2006;48(1):39‐42. 10.4103/0019-5545.31617 20703413 PMC2913641

[2] O'Brien P , Oyebode F . Psychotropic medication and the heart. Adv Psych Treat. 2003;9(6):414‐423. 10.1192/apt.9.6.414

[3] Ioannou A , Perera D . Delayed olanzapine induced myocarditis and rhabdomyolysis. Postgrad Med J. 2022;98(Issue 1165):824. 10.1136/postgradmedj-2021-141118 34625500

[4] Hilli J , Laine K . Can olanzapine cause cardiomyopathy? Sweden (SE): Drugle. Swedish Institute for Drug Informatics; 2012.

[5] Dukes MNG , Aronson JK . Meyler's Side Effects of Drugs. 14th ed. 2000 Elsevier.

[6] Lee TW , Tsai SJ , Hwang JP . Severe cardiovascular side effects of olanzapine in an elderly patient: case report. Int J Psych Med. 2003;33(4):399‐401. 10.2190/U99G-XDML-0GRG-BYE0 15152790

[7] Berstein JG . Management of psychotropic drug induced obesity. In: Bjorntrop P , Brodoff BN , eds. Obesity. Philadelphia, 1992.

[8] Guille C , Sachs GS , Ghaemi SN . A naturalistic comparison of clozapine, risperidone, and olanzapine in the treatment of bipolar disorder. J Clin Psychiatry. 2000;61(9):638‐642. 10.4088/jcp.v61n0907 11030483

[9] Farver D . Weight gain and longterm complications with atypical antipsychotics. S D J Med. 2001;54(1):11‐12. 11211419

[10] Wang J , Liu Y , Zhu W , Zhang F , Zhou Z . Olanzapine‐induced weight gain plays a key role in the potential cardiovascular risk: evidence from heart rate variability analysis. Sci Rep. 2014;4:7394. 10.1038/srep07394 25487560 PMC4260225

[11] Citrome L , Collins JM , Nordstrom BL , et al. Incidence of cardiovascular outcomes and diabetes mellitus among users of second‐generation antipsychotics. J Clin Psychiatry. 2013;74(12):1199‐1206. 10.4088/JCP.13m08642 24434088

[12] Wang J , Liu Y , Zhu W , Zhang F , Zhou Z . Olanzapine‐induced weight gain plays a key role in the potential cardiovascular risk: evidence from heart rate variability analysis. Sci Rep. 2014;4:7394. 10.1038/srep07394 25487560 PMC4260225

[13] Melkersson KI , Hulting AL , Brismar KE . Elevated levels of insulin, leptin, and blood lipids in olanzapine‐treated patients with schizophrenia or related psychoses. J Clin Psychiatry. 2000;61(10):742‐749. 10.4088/jcp.v61n1006 11078035

[14] Nemeroff CB . Dosing the antipsychotic medication olanzapine. J Clin Psychiatry. 1997;58(suppl 10):45‐49. 9265916

[15] Allison DB , Mentore JL , Heo M , et al. Antipsychotic‐induced weight gain: a comprehensive research synthesis. Am J Psychiatry. 1999;156(11):1686‐1696. 10.1176/ajp.156.11.1686 10553730

[16] Puttegowda B , Theodore J , Basappa R , Nanjappa MC . Olanzapine induced dilated cardiomyopathy. Malaysian J Med Sci. 2016;23(2):82‐84. PMC4976704 27547120

[17] Aune D , Sen A , Norat T , et al. Body mass index, abdominal fatness, and heart failure incidence and mortality: a systematic review and dose‐response meta‐analysis of prospective studies. Circulation. 2016;133:639‐649. 26746176 10.1161/CIRCULATIONAHA.115.016801

[18] Shen Q , Hiebert JB , Rahman FK , Krueger KJ , Gupta B , Pierce JD . Understanding Obesity‐Related high output heart failure and its implications. Int J Heart Fail. 2021;3(3):160‐171. 10.36628/ijhf.2020.0047 36262639 PMC9536652

[19] Singh S , Sharma S . High‐Output Cardiac Failure. StatPearls. StatPearls Publishing; 2023. 30020709

[20] Turer AT , Hill JA , Elmquist JK , Scherer PE . Adipose tissue biology and cardiomyopathy: translational implications. Circ Res. 2012;111(12):1565‐1577. 10.1161/CIRCRESAHA.111.262493 23223931 PMC3532954

[21] Kolwicz SC , Purohit S , Tian R . Cardiac metabolism and its interactions with contraction, growth, and survival of cardiomyocytes. Circ Res. 2013;113(5):603‐616. 10.1161/CIRCRESAHA.113.302095 23948585 PMC3845521

[22] Gibb AA , Hill BG . Metabolic coordination of physiological and pathological cardiac remodeling. Circ Res. 2018;123(1):107‐128. 10.1161/CIRCRESAHA.118.312017 29929976 PMC6023588

[23] Lavie CJ , Alpert MA , Arena R , Mehra MR , Milani RV , Ventura HO . Impact of obesity and the obesity paradox on prevalence and prognosis in heart failure. JACC Heart Fail. 2013;1(2):93‐102. 10.1016/j.jchf.2013.01.006 24621833

[24] Mishra S , Kass DA . Cellular and molecular pathobiology of heart failure with preserved ejection fraction. Nat Rev Cardiol. 2021;18(6):400‐423. 10.1038/s41569-020-00480-6 33432192 PMC8574228

[25] Yazıcı D , Sezer H . Insulin resistance, obesity and lipotoxicity. Adv Exp Med Biol. 2017;960:277‐304. 10.1007/978-3-319-48382-5_12 28585204

[26] Syme C , Shin J , Richer L , Gaudet D , Paus T , Pausova Z . Sex differences in blood pressure hemodynamics in middle‐aged adults with overweight and obesity. Hypertension. 2019;74(2):407‐412. 10.1161/HYPERTENSIONAHA.119.13058 31230538

[27] Sainju NK , Shah RK , Joshi SK . Screening for hypertension and obesity in rural population of Nepal. Kathmandu Univ Med J (KUMJ). 2018;16(61):4‐7. 30631008

[28] DeMarco VG , Aroor AR , Sowers JR . The pathophysiology of hypertension in patients with obesity. Nat Rev Endocrinol. 2014;10(6):364‐376. 10.1038/nrendo.2014.44 24732974 PMC4308954

[29] Grassi G , Mark A , Esler M . The sympathetic nervous system alterations in human hypertension. Circ Res. 2015;116(6):976‐990. 10.1161/CIRCRESAHA.116.303604 25767284 PMC4367954

[30] Van Gaal LF , Maggioni AP . Overweight, obesity, and outcomes: fat mass and beyond. The Lancet. 2014;383(9921):935‐936. 10.1016/S0140-6736(13)62076-0 24269110

[31] Chirinos JA , Segers P , Hughes T , Townsend R . Large‐Artery stiffness in health and disease. J Am Coll Cardiol. 2019;74(9):1237‐1263. 10.1016/j.jacc.2019.07.012 31466622 PMC6719727

[32] Weber T , Chirinos JA . Pulsatile arterial haemodynamics in heart failure. Eur Heart J. 2018;39(43):3847‐3854. 10.1093/eurheartj/ehy346 29947746 PMC6234849

[33] Wildman RP , Mackey RH , Bostom A , Thompson T , Sutton‐Tyrrell K . Measures of obesity are associated with vascular stiffness in young and older adults. Hypertension. 2003;42(4):468‐473. 10.1161/01.HYP.0000090360.78539.CD 12953016

[34] Weisbrod RM , Shiang T , Al Sayah L , et al. Arterial stiffening precedes systolic hypertension in diet‐induced obesity. Hypertension. 2013;62(6):1105‐1110. 10.1161/HYPERTENSIONAHA.113.01744 24060894 PMC3951434

[35] Aggoun Y , Farpour‐Lambert NJ , Marchand LM , Golay E , Maggio ABR , Beghetti M . Impaired endothelial and smooth muscle functions and arterial stiffness appear before puberty in obese children and are associated with elevated ambulatory blood pressure. Eur Heart J. 2008;29(6):792‐799. 10.1093/eurheartj/ehm633 18245115

[36] Kaess BM , Rong J , Larson MG , et al. Aortic stiffness, blood pressure progression, and incident hypertension. JAMA. 2012;308(9):875‐881. 10.1001/2012.jama.10503 22948697 PMC3594687

[37] Saxena T , Ali AO , Saxena M . Pathophysiology of essential hypertension: an update. Expert Rev Cardiovasc Ther. 2018;16(12):879‐887. 10.1080/14779072.2018.1540301 30354851

[38] Obokata M , Reddy YNV , Pislaru SV , Melenovsky V , Borlaug BA . Evidence supporting the existence of a distinct obese phenotype of heart failure with preserved ejection fraction. Circulation. 2017;136(1):6‐19. 10.1161/CIRCULATIONAHA.116.026807 28381470 PMC5501170

[39] Olivier A , Pitt B , Girerd N , et al. Effect of eplerenone in patients with heart failure and reduced ejection fraction: potential effect modification by abdominal obesity. insight from the EMPHASIS‐HF trial. Eur J Heart Fail. 2017;19(9):1186‐1197. 10.1002/ejhf.792 28303624

[40] Packer M . Leptin‐Aldosterone‐Neprilysin axis: identification of its distinctive role in the pathogenesis of the three phenotypes of heart failure in people with obesity. Circulation. 2018;137(15):1614‐1631. 10.1161/CIRCULATIONAHA.117.032474 29632154

[41] Rosengren A , Åberg M , Robertson J , et al. Body weight in adolescence and long‐term risk of early heart failure in adulthood among men in Sweden. Eur Heart J. 2016;38(24):ehw221. 10.1093/eurheartj/ehw221 PMC5837553 27311731

[42] Cerit L , Kemal H , Gunsel A , Duygu H . High‐output heart failure. J Am Coll Cardiol. 2017;69(1):112‐113. 10.1016/j.jacc.2016.08.080 28057238

[43] Reddy YNV , Melenovsky V , Redfield MM , Nishimura RA , Borlaug BA . High‐output heart failure. J Am Coll Cardiol. 2016;68(5):473‐482. 10.1016/j.jacc.2016.05.043 27470455

[44] Barzizza F . [Obesity and the heart]. Minerva Gastroenterol Dietol. 2001;47(4):229‐234. 16493382

[45] Friedman SE , Andrus BW . Obesity and pulmonary hypertension: a review of pathophysiologic mechanisms. J Obes. 2012;2012:505274. 10.1155/2012/505274 22988490 PMC3439985

[46] Mair KM , Harvey KY , Henry AD , Hillyard DZ , Nilsen M , MacLean MR . Obesity alters oestrogen metabolism and contributes to pulmonary arterial hypertension. Eur Respir J. 2019;53(6):1801524. 10.1183/13993003.01524-2018 30923189 PMC6581204

[47] Senaratna CV , Perret JL , Lodge CJ , et al. Prevalence of obstructive sleep apnea in the general population: a systematic review. Sleep Med Rev. 2017;34:70‐81. 10.1016/j.smrv.2016.07.002 27568340

[48] Warricker F , Islam Z , Shah BN . Lesson of the month 1: obesity hypoventilation (Pickwickian) syndrome: a reversible cause of severe pulmonary hypertension. Clin Med. 2017;17(6):578‐581. 10.7861/clinmedicine.17-6-578 PMC6297698 29196363

[49] Ray I , Mahata SK , De RK . Obesity: an immunometabolic perspective. Front Endocrinol. 2016;7:157. 10.3389/fendo.2016.00157 PMC5149556 28018292

[50] Coulter DM . Antipsychotic drugs and heart muscle disorder in international pharmacovigilance: data mining study. BMJ. 2001;322(7296):1207‐1209. 10.1136/bmj.322.7296.1207 11358771 PMC31617

[51] Frühbeck G , Méndez‐Giménez L , Fernández‐Formoso JA , Fernández S , Rodríguez A . Regulation of adipocyte lipolysis. Nutr Res Rev. 2014;27(1):63‐93. 10.1017/S095442241400002X 24872083

[52] Song Z , Xiaoli A , Yang F . Regulation and metabolic significance of *De Novo* lipogenesis in adipose tissues. Nutrients. 2018;10(10):1383. 10.3390/nu10101383 30274245 PMC6213738

[53] Belhani D , Frassati D , Mégard R , et al. Cardiac lesions induced by neuroleptic drugs in the rabbit. Exp Toxicol Pathol. 2006;57(3):207‐212. 10.1016/j.etp.2005.09.003 16410188

[54] Clapham E , Reutfors J , Linder M , Brandt L , Sundström J , Bodén R . The association between exposure to clozapine, olanzapine, and quetiapine and the outcomes perimyocarditis and heart failure: A population‐based cohort study. Psychiatry Res. 2023;326:115336. 10.1016/j.psychres.2023.115336 37451082

[55] Li XQ , Tang XR , Li LL . Antipsychotics cardiotoxicity: what's known and what's next. World J Psychiatry. 2021;11(10):736‐753. 10.5498/wjp.v11.i10.736 34733639 PMC8546771

[56] Shamshoum H , McKie GL , Medak KD , Ashworth KE , Kemp BE , Wright DC . Voluntary physical activity protects against olanzapine‐induced hyperglycemia. J Appl Physiol. 2021;130(2):466‐478. 10.1152/japplphysiol.00876.2020 33382959

